# Pathophysiology and Emerging Molecular Therapeutic Targets in Heterotopic Ossification

**DOI:** 10.3390/ijms23136983

**Published:** 2022-06-23

**Authors:** Favour Felix-Ilemhenbhio, George A. E. Pickering, Endre Kiss-Toth, Jeremy Mark Wilkinson

**Affiliations:** 1 Department of Oncology and Metabolism, The Medical School, University of Sheffield, Beech Hill Road, Sheffield S10 2RX, UK; ffelixilemhenbhio1@sheffield.ac.uk (F.F.-I.); georgeaepickering@gmail.com (G.A.E.P.); 2 Department of Infection, Immunity and Cardiovascular Disease, University of Sheffield, Sheffield S10 2RX, UK; e.kiss-toth@sheffield.ac.uk

**Keywords:** heterotopic ossification, genetics, bone morphogenetic protein, activin A/ALK2, retinoic acid receptor, Hoxa11+ mesenchymal stromal cells

## Abstract

The term heterotopic ossification (HO) describes bone formation in tissues where bone is normally not present. Musculoskeletal trauma induces signalling events that in turn trigger cells, probably of mesenchymal origin, to differentiate into bone. The aetiology of HO includes extremely rare but severe, generalised and fatal monogenic forms of the disease; and as a common complex disorder in response to musculoskeletal, neurological or burn trauma. The resulting bone forms through a combination of endochondral and intramembranous ossification, depending on the aetiology, initiating stimulus and affected tissue. Given the heterogeneity of the disease, many cell types and biological pathways have been studied in efforts to find effective therapeutic strategies for the disorder. Cells of mesenchymal, haematopoietic and neuroectodermal lineages have all been implicated in the pathogenesis of HO, and the emerging dominant signalling pathways are thought to occur through the bone morphogenetic proteins (BMP), mammalian target of rapamycin (mTOR), and retinoic acid receptor pathways. Increased understanding of these disease mechanisms has resulted in the emergence of several novel investigational therapeutic avenues, including palovarotene and other retinoic acid receptor agonists and activin A inhibitors that target both canonical and non-canonical signalling downstream of the BMP type 1 receptor. In this article we aim to illustrate the key cellular and molecular mechanisms involved in the pathogenesis of HO and outline recent advances in emerging molecular therapies to treat and prevent HO that have had early success in the monogenic disease and are currently being explored in the common complex forms of HO.

## 1. Introduction

Heterotopic ossification (HO) is a disorder characterised by bone development within tissues where bone does not normally exist. Several presentations of HO have been described since its early documentation in 1883 by Riedel and its first association with musculoskeletal trauma in World War One combatants in 1918 [1]. There are two forms of HO traditionally described: the rare ‘genetic disease’, and the more common acquired, or ‘post-traumatic’, HO. The monogenic HO diseases, which follow a Mendelian pattern of inheritance, include fibrodysplasia ossificans progressiva (FOP) and progressive ossific heteroplasia (POH). FOP is a rare debilitating disease with a prevalence of 1–2 cases per million persons in which muscle and connective tissues are gradually substituted by bone that is commonly triggered by minor trauma events [2,3]. POH is an extremely rare disease affecting less than 60 people worldwide, ref. [4,5] in which ossification develops initially in the deeper layers of the dermis and subcutaneous fat and spreads to include muscle and tendons as the disease progresses. Both diseases are associated with progressive disability and early death [6]. The term acquired, or “post-traumatic” HO describes extra-skeletal bone formation that occurs following musculoskeletal or neurological trauma and burns [7]. Acquired HO occurs in 20–30% of patients with spinal cord injury [8], 10–20% of patients with closed head injury [8], up to 50% of patients after total hip replacement [9], and up to 70% of patients following high-energy combat trauma [10].

The present review provides an overview of our current understanding of the molecular biology of HO initiation and development, including the cellular and genetic origins of HO. Based on these molecular advances in our understanding of the disease, we also review the current status of evolving molecular therapies for HO prevention and treatment. Throughout the article, we use the term HO to describe acquired HO and the terms FOP and POH to describe the specific monogenic disorders.

## 2. Overview of Normal Bone Formation

In order to understand the mechanisms of bone formation in HO, a brief review of normal bone formation is given against which HO development will be compared. Normal mature bone is formed through one of two mechanisms, termed intramembranous and endochondral ossification. The progenitor cell for both processes is the mesenchymal precursor, but the mechanism and site at which ossification occurs differs (reviewed in [11,12]). In intramembranous ossification, a sheet of mesenchymal connective tissue, termed the fibrous membrane, forms the template of the future bone. Bones forming through this mechanism are typically flat, including the cranium, sternum, ribs, and scapula. The mesenchymal precursor cells differentiate into osteoblasts or into supporting blood vessels. The osteoblasts secrete osteoid, an extracellular matrix comprising collagen and other organic proteins that entraps the osteoblasts as the osteoid mineralises. Once entrapped, the osteoblasts trans-differentiate into osteocytes that remain as mechanosensing cells within the bone matrix. Osteoblasts on the surface of the bone transdifferentiate to form a cellular layer termed the periosteum. The periosteum is responsible for cortical bone synthesis, and envelopes the cancellous bone that is continuous with the haematopoietic red bone marrow. In endochondral ossification, bone formation occurs through an intermediate, cartilaginous stage that serves as a template for the final bone. The long bones, including the clavicle, humerus, radius, ulna, metacarpals, phalanges, femur, fibula, tibia, metatarsals, and phalanges form through endochondral ossification. The process commences as mesenchymal stem cells condense and differentiate into chondrocytes to form the cartilage template. This is followed by hypertrophy and subsequent apoptosis of the central cells, whilst mesenchymal progenitors at the template surface differentiate into osteoblasts and osteoclasts. The hypertrophic and apoptotic cartilage core is innervated, vascularised, and replaced by bone and bone marrow in the primary ossification centre. At the developing bone metaphysis, a hypertrophic component of the growing cartilage is constantly substituted by trabecular bone to mediate longitudinal bone growth. The non-vascularised cartilage at the ends of the bone is invaded by epiphyseal vessels to initiate the secondary ossification centre. Between the epiphyseal and metaphyseal bone centres reside layers of chondrocytes that form growth plates to further support longitudinal growth. Longitudinal growth stops as the growth plate is fully resorbed to leave a single marrow cavity within the long bone.

## 3. Cellular Origins of HO

In HO development, following the initiating stimulus bone may form within a range of extraosseous tissues of mesenchymal origin and may involve either of the above mechanisms of bone formation. The architectural features of HO resemble normal bone and include a zonal mineralization pattern, mature cortical bone at the periphery, and a central marrow component [13]. Chalmers et al. (1975) first proposed the basic common requirements for HO formation: osteogenic precursor cells, a permissive environment and an inducing agent [14]. This model is consistent with HO formation through either the endochondral or intramembranous routes. A summary of current concepts of the cellular origins of HO is given below and is reviewed further elsewhere [15,16].

### 3.1. Hematopoietic Cells

In the 1970s, hematopoietic stem cells or other precursors recruited to the lesions from bone marrow were suggested to contribute to the induction and formation of ectopic bone in patients with FOP [17]. Lymphocytes taken from FOP patients were subsequently shown to overexpress Bone Morphogenetic Protein 4 (BMP4), a potent bone-inducing protein [18]. Olmsted-Davis and colleagues investigated hematopoietic side-population (SP) cells as possible precursors for HO [19]. These cells were known to possess multi-lineage potential, with the ability to differentiate into skeletal myocytes [20] and vascular endothelial cells [21]. SP cells were isolated from the bone marrow of C57BL/6 CD45.2 Rosa26 mice and their osteogenic potential was tested by transplantation into C57BL/6 CD45.1 mice. Both osteoblasts and osteocytes from the subsequent newly formed bone stained positively for markers of donors SP cells indicating osteogenic potential [19]. Dominici and colleagues demonstrated in Friend leukemia virus B/ NIH Jackson (FVB/NJ) mice that transplantable fluorescently-labelled marrow cells from the non-adherent population can produce functional osteoblasts, osteocytes and hematopoietic cells [22]. Kaplan and colleagues observed in a patient with FOP that bone marrow transplantation for treating anaemia was not sufficient to inhibit FOP, but that pharmacological suppression of the donor’s immune system following transplantation inhibited FOP [23]. In mice, hematopoietic cells contributed to the inflammatory and bone marrow-repopulating stages of BMP4-induced HO by recruiting and activating osteogenic precursors, but they did not act directly as a cellular precursor of HO [23]. These findings contrast with those of Otsuru and colleagues who showed a contribution of hematopoietic cells to bone formation in BMP2-induced intramuscular HO, although only a minority of bone-marrow derived cells were embedded in the definitive heterotopic bone [24,25]. More recently, analysis of clinical tissue following musculoskeletal injury in humans has demonstrated that circulating osteogenic progenitor cells of bone marrow origin, characterised by both type 1 collagen and CD45 immunopositivity, are found in early fibroproliferative and neovascular HO lesions, supporting the concept that circulating mononuclear progenitors can seed inflammatory sites to initiate HO formation [26]. Taken together these studies suggest that haematopoietic cells of bone marrow origin contribute to both FOP and acquired HO development most likely through their creation of the necessary pro-osteogenic environment, but are unlikely to be significant direct osteogenic progenitors.

### 3.2. Endothelial Cells

Vascular endothelial cells have been suggested as a primary candidate for HO formation due to their multilineage potential via endothelial-mesenchymal transition (EndMT) and the expression of endothelial markers in FOP lesions [27,28]. During EndMT, endothelial cells loose cell-cell adhesion and change polarity, reconfiguring into a spindle-shape, and reducing the expression of endothelial markers whilst increasing mesenchymal marker expression. Following transition, these cells are highly motile and invasive and play an important role in both tissue development and disease [29,30,31]. Medici and colleagues showed in vitro that endothelial cells over-expressing Activin Receptor-like Kinase 2 (ALK2, also called ACRV1), or treated with the ALK2 ligands TGF-β2 or BMP4, can dedifferentiate into stem cells with the capacity to re-differentiate into cartilage or bone cells [28]. In vivo data in the neuron-specific enolase-BMP4 (NSE-BMP4) mouse also show that ectopic cartilage and bone cells express endothelial biomarkers such as vWF, VE-cadherin, Tie1, and Tie2 after injection of purified BMP [27,28], after transgenic over-expression of ALK2 [28], or after muscle injury [27]. Tie2 and vWF are also expressed in chondrogenic and osteogenic lesions from FOP patients, whereas osteoblasts and chondrocytes from normal cartilage or bone do not express these biomarkers [28]. Lineage tracing in Tie2-Cre transgenic mice found that 50% of the cartilage and bone cells in HO lesions were of endothelial origin [27,28]. However, CD31+ endothelial cells were shown to not contribute to heterotopic cartilage or bone formation directly in the mouse following intramuscular BMP2 injection, but they did participate in lesion angiogenesis [32] and to HO development following burn/tenotomy injury [33]. The different outcomes in the last two studies may be attributed to differences in the Cre drivers or in the HO models used [16]. A further limitation of the lineage trace studies is that the markers expressed by endothelial cells can also be expressed by other cell types. Tie2, which is expressed in endothelial cells to regulate development and maintenance of vasculature [34], is also expressed in hematopoietic cells [35,36], and by a population of Tie2+ PDGFRα+ Sca1+ multipotent mesenchymal progenitors that was shown to contribute to HO initiation [32]. Furthermore, musculoskeletal injury induces expression of endothelial markers Tie2, CD31 and VE-cadherin in mesenchymal, non-endothelial cells [33]. Taking together, the studies outlined above suggest that endothelial cells can undergo EndMT to initiate HO but they are unlikely to be pivotal, as Tie2+, CD31+ or VeCadherin+ progenitors also arise from other cell types and vary with the HO induction model used. These inconsistencies underscore the idea that the cellular populations contributing to HO development are highly tissue and context-specific.

### 3.3. Fibro-Adipogenic Cells

Fibro-adipogenic precursors (FAPs) are a population of PDGFRα+ SCA1+ multipotent cells located within, but not exclusive to, skeletal muscle [32,37,38]. FAPs are found near vascular elements, but are unlike pericytes in that they do not share a basal lamina with the endothelium and are NG2- [32,38]. Muscle resident FAPs support muscle regeneration but lack myogenic potential [29,37,38]. FAPs were first discovered due to their fibrogenic and adipogenic capacities [37,38]. They were later shown to possess osteogenic potential when stimulated with BMP in culture and in vivo [32]. Wosczyna and colleagues observed that Tie2-Cre lineage labelled FAPs made up ~50% of heterotopic bone and cartilage in the mouse [32]. These cells have been proposed to play a major role in human FOP [39,40]. Several studies in the mouse show that progenitors of intramuscular and intratendinous HO are frequently PDGFRα+ and positive for cartilage and bone formation markers [38,39,41,42,43,44]. Using a mouse FOP model in which *ACVR1* (that encodes ALK2) was genetically manipulated, Dey and colleagues showed that FAP-like cells can be divided into two lineages, Scx+ tendon-derived progenitors and a muscle-resident interstitial Mx1+ population [39]. The Scx+ progenitors mediated endochondral HO without exogenous injury, whilst the Mx1+ population mediated injury-dependent HO. PDGFRα+ cells made up a minor subgroup of Mx1+ and Scx+ lineages; however, constitutive activation of *ACVR1* signalling demonstrated that PDGFRα+ subsets had an enhanced osteogenic and chondrogenic potential compared to unfractionated Scx+ or Mx1+ cells. Eisner and colleagues demonstrated that tissue resident FAPs in skeletal muscle are the primary source of osteogenic cells in the murine BMP2-Matrigel model of post-traumatic HO [44]. In the same study using Notexin to induce muscle damage, they demonstrated that FAPs contribute to the formation of mature bone without the addition of exogenous BMP2. Moreover, when FAPs were cleared by macrophages at day 3–4 after injury, osteogenic genes were downregulated. Taken together, these findings suggest that FAPs can contribute to most HO presentations due to their broad distribution across tissue types and their documented presence in HO and that cells of hematopoietic origin play a role in stimulating their osteogenic potential.

### 3.4. Myosatellite Cells

Myosatellite cells are myogenic muscle-resident stem cells that are pivotal in skeletal muscle regeneration [45]. They are located between the myofibre sarcolemma and basal lamina, and give rise to myodifferentiated cells following muscle injury [46]. They were initially considered a primary precursor for muscle HO due to their osteogenic potential in culture in response to BMPs in the C2C12 murine myoblast model [47] and in human myogenic progenitor cells [48]. However, lineage and transplantation studies indicate that they contribute minimally to BMP-induced HO in vivo [27,32,49,50]. Further, targeted expression of constitutively-activated *ACVR1*/*ALK2* (caACVR1) [39,51] and *ACVR1* (R206H) [40] in myosatellite cells is insufficient to induce HO. Although Lees-Shephard and Goldhamer [16] have proposed that myosatellite cells do not contribute to HO initiation, several lines of in vivo data do support their role in its pathogenesis. BMP signalling is a primary mechanism leading to the formation of acquired and genetic HO and has also been associated with the physiological regulation of skeletal muscle mass [52]. When transplanted into the quadriceps of nude mice, skeletal muscle myoblasts have been shown to promote osteogenic differentiation [53]. Muscle-derived stem cells express BMP4 and differentiate into bone [54]. BMPs at the location of muscle injury inhibit myogenesis and promote osteogenesis of myoblasts, both in vitro [47] and in vivo [32]. Further, serum taken from animals following a burn injury increases the osteogenic capacity of myosatellite cells, suggesting a role in burn-induced HO [55]. Taken together, these findings indicate that the muscle tissue provides a permissive environment for HO and that following musculoskeletal trauma BMPs can modulate endogenous muscle progenitors to form heterotopic bone.

### 3.5. Other Cell Types

Through in vivo models, several other progenitor cell types have also been identified, including pericytes, tendon and ligament progenitors, and transient brown adipocyte-like cells (Table 1). Although these cell types are associated with HO initiation, their precise contributions remain unclear. More recently, using a burn/tenotomy injury in *Hoxa11-CreER^T2^*; *ROSA-LSL-TdTomato* mice, Pagani and colleagues have traced the cell fate of MSCs in HO development using single-cell sequencing [56]. They found that MSCs of the *Hoxa11* lineage differentiate through both the endochondral and osteogenic route into HO bone in the mouse forelimb following burn/tenotomy injury. During HO progression, the *Hoxa11*-lineage cells expressed transcriptional profiles characteristic of both osteogenesis and chondrogenesis. Previous studies have shown that *Hoxa11+* multipotent stromal cells are self-renewing and persistent throughout the life of mice, and that Hoxa11 contributes to bone formation, maintenance and repair [57,58,59].

**Table 1:** Overview of cell types investigated for their contribution to heterotopic ossification.

Cell Type	Location	Description	Key Papers
Hematopoietic cells	Bone marrow	Contribute to inflammation and marrow-repopulating stages. Contribution to HO is unclear.	[19,23,25,60]
Endothelial cells	Blood and lymphatic vessels	Contribute to HO through EndMT route, but may be overestimated due to lack of surface marker endothelial cell-specificity.	[28,35]
FAPs	Muscle and related soft tissues; widely spread in other tissues	Support muscle regeneration. Contribute to a high percentage of HO.	[32,43,61]
Myosatellite cells	Muscle	BMP2-induced HO. Contribution low based on most lineage studies.	[32,48]
Pericytes	Vascular basement membrane	BMP-induced HO but assessment of contribution unclear due to high degree of heterogeneity.	[50,62,63,64]
Hoxa11+ Mesenchymal stromal cells	Tendon, muscle and skeletal tissue	Contribute to skeletal repair, express chondrogenic and osteogenic transcription profile following injury.	[56,57,58,59]
Tendon and ligament progenitor cells	Tendon Ligament	Account for 25 and 40% of heterotopic bone and cartilage, respectively, after bone/tendonectomy based on Scx-Cre labelling. Molecularly heterogeneous.	[39,43,65]
Sensory neurons	Dermis, epidermis, and muscle spindle	Mediate HO formation via substance P and calcitonin gene-related peptide. BMP2 may induce neurogenic inflammation to remodel nerve and release HO precursor cells. May explain how HO occurs following traumatic brain injury. Mice lacking sensory neurons cells do not develop HO. Tie2+ endoneurial progenitors the major HO cell contributors in a mice model; however, Tie2 marker is also expressed in endothelial and mesenchymal cells.	[66,67,68,69]
Transient brown adipocyte-like cells	Adipose	Specialized pool of brown adipocytes that contribute to HO. Associated to deposition of cartilage. Detected in human traumatic injury-induced HO.	[70,71]

Due to the heterogenic nature of HO aetiology, several cell types contribute depending on the site and initiating factors. This raises the issue of which cell and experimental model is most appropriate for investigating the function of HO susceptibility genes in culture and/or in vivo. A conclusive answer to this question remains elusive, nevertheless, the role of specific genes may be best examined by investigating how they affect the signalling response of precursor cells to promote bone formation and/or maintenance using an experimental model most appropriate to the type of HO investigated.

## 4. Signalling Pathways in HO

### 4.1. BMP Signalling

BMPs are a family of signalling molecules that belong to the Transforming Growth Factor-β (TGF-β) superfamily of proteins. Discovered by Urist in 1965 [72], they play a crucial role in bone formation and repair, and in HO development [73]. During normal bone development and physiological homeostasis, BMP ligands bind to a heterotetrameric complex of two BMPRI and two BMPRII transmembrane serine/threonine kinase receptors to initiate chondrogenesis and osteogenesis. The BMPs that initiate signalling through this mechanism and the osteogenic processes that they initiate are summarised in Table 2.

**Table 2:** Overview of BMPs and their role in major cellular process and heterotopic ossification.

Signalling Protein	Function	Key Papers
BMP1	Bone formation and homeostasis.	[74]
BMP2	Induces bone and cartilage development. Induces EndMT transition. Also involved in hedgehog pathway, cardiac cell differentiation, embryonic development.	[75,76,77,78]
BMP3	Bone and cartilage development; antagonizes other BMPs in osteo-differentiation.	[79]
BMP4	Potently induces chondro- and osteogenic differentiation; induces EndMT transition. Also involved in embryonic development, adipogenesis, neurogenesis.	[80,81,82,83]
BMP5	Bone and cartilage development; may play a role in some cancer types; expressed in the visual apparatus.	[84,85,86]
BMP6	Osteogenic differentiation; closely related to BMP5 and BMP7; regulates iron metabolism	[87,88,89]
BMP7	Bone homeostasis; induces osteoblast differentiation through SMAD canonical pathway; involved in embryonic development, adipogenesis.	[90,91,92]
BMP8	Expressed in developing skeleton; osteogenesis and germ cell generation.	[93,94,95,96]
BMP9/GDF2	Induces chondro- and osteogenesis; cannot be blocked by BMP3 unlike most BMPs; involved in lymphatic development.	[97,98,99]
BMP10	Involved in the trabeculation oof the heart and regulates monocyte recruitment to the vascular endothelium.	[100,101,102]
BMP11/GDF11	Augments bone formation; induces embryonic development.	[103,104]
BMP12/GDF7	Inhibits endochondral bone growth; induces tenogenic differentiation; regulates bone structure	[105]
BMP13/GDF6/CDMP2	Establishes the boundaries between skeletal elements during development; induces tenogenic differentiation	[105,106]
BMP14/GDF5/CDMP1	Regulates skeletal development and joint formation; promotes fracture healing.	[106,107,108]
BMP15	Involved in fertilization and ovulation	[109,110]

**Figure 1:**
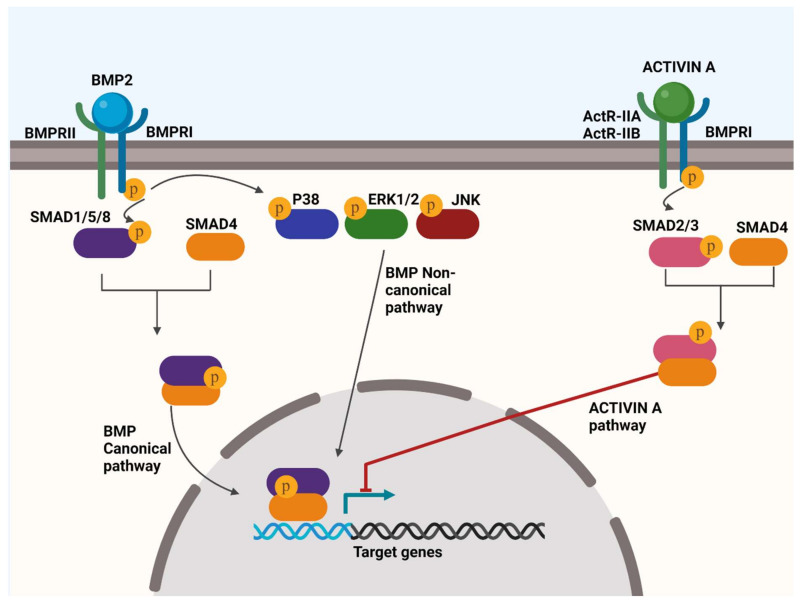
BMP receptor activation and downstream signalling and its antagonism through the Activin A pathway. In the canonical pathway, SMAD1/5/8 is activated and interacts with SMAD4 to promote expression of target genes that induce bone formation. In the non-canonical SMAD pathway, p38 MAPK, ERK1/2 and/or JNK are activated to promote the expression of osteogenic target genes. BMP signalling is antagonised by the binding of Activin A to its receptor complex to initiate SMAD2/3 signalling that acts to suppress BMP target gene transcriptional activation.

Four type I BMP receptors (ALK1, ALK2 (also termed ACVR1), ALK3 and ALK6) bind BMP ligands. Three receptors (BMPR2, ALK4 and ALK7) serve as type II BMP receptors. ALK4 and ALK7 (also termed ActR-IIA and ActR-IIB), also act as receptors for activins, whilst BMPR2 only binds BMPs (Figure 1).

Downstream signalling following BMP receptor activation occurs through 2 distinct pathways: 1. SMAD canonical pathways, in which SMAD 1/5/8 proteins are phosphorylated to promote expression of chondro- or osteogenic genes [111]; 2. Non-canonical SMAD pathways where p38 MAPK, ERK or JNK are activated [112,113,114,115]. Under normal physiological conditions, these chondro- and osteogenic signalling pathways are antagonised by Activin A (another TGF-β superfamily member) binding to a heterotetrameric receptor complex comprising two ActR BMPRII receptors and two BMPRI receptors to initiate SMAD2/3 phosphorylation and downstream signalling as a negative feedback mechanism for gene transcriptional activation that is initiated by BMP signalling [116]. These pathways should not be viewed as independent, as crosstalk between them occurs [117,118,119,120]. BMP2 is overexpressed in clinically evolving HO tissue after trauma [121,122]. Augmented BMP signalling also occurs following trauma-induced HO development in animal models whilst BMP antagonism reduces HO severity [123,124]. Experimental models of HO therefore commonly use exogenous BMP2 [27,68] or overexpression of BMP4 [125], or recombinant BMP2 (rhBMP2) [27,126] as the HO initiator. BMP signalling is also a key feature of the heritable forms of the disease [127]. In FOP, a mutation in *ACVR1* that encodes the BMP type 1 receptor ALK2, causes its constitutive activation, initiating downstream BMP signalling regardless of BMP ligand binding [124].

### 4.2. mTOR Signalling

**Figure 2:**
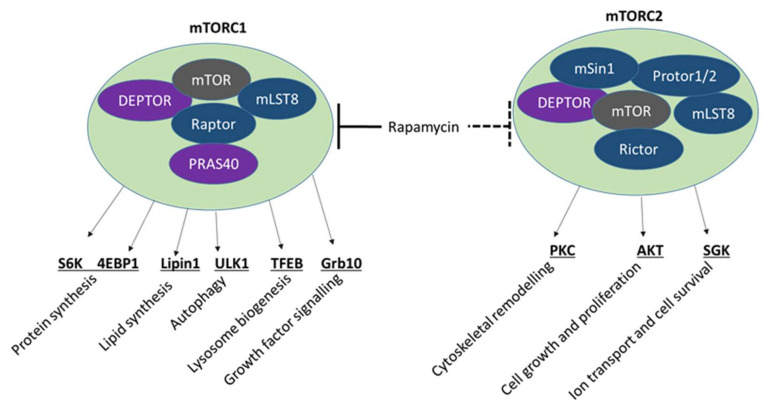
mTOR signalling pathway. Rapamycin inhibits mTORC1 and mTORC2, which in turn modulate several downstream osteogenic pathways. Acute rapamycin treatment inhibits mTORC1 whilst repeated dosing of rapamycin also inhibits mTORC2. Both mTORC1 and mTORC2 are activated by Wnt and IGF. mTORC1 is also activated by BMP2 and mTORC2 is also activated by mechanical and chemical signals to promote osteogenesis.

The mammalian target of rapamycin (mTOR) signalling pathway is involved in several cellular processes, including chondrogenesis, osteogenesis and skeletal development [128,129]. The FOP activating mutation in *ACVR1* has been shown to increase mTOR signalling [130]. Conversely, rapamycin suppresses bone formation in experimental models for FOP [41,130], trauma-induced HO [41,131], and in leptin-induced osteogenesis in both in vitro and in vivo models [132] through inhibition of mTOR complexes mTORC1 and mTORC2 [133] (Figure 2). Rapamycin is currently being studied in a phase 2 clinical trial (UMIN000028429) of the disease. BMP2 also promotes osteogenesis through an mTORC1-dependent mechanism [134], whilst mTORC2 modulates osteogenesis in response to a range of mechanical or chemical cues [128,135,136].

### 4.3. Other Signalling Pathways

Hypoxia-inducible factors (HIFs) activate genes that mediate adaptive responses to reduced oxygen tension [137,138]. HIFs augment HO formation [41] and couple bone and vascular growth during development [138]. Retinoic acid receptor (RAR) signalling is mediated by retinoids (metabolic derivatives of vitamin A), which are potent morphogens that promote both chondro- and osteogenesis to shape skeletal development [139]. In retinoic acid (RA) mediated gene activation, RA binds to a heterodimer complex comprising RAR and the retinoid X receptor (RAR-RXR). RAR-RXR then activates gene transcription by binding to DNA motifs termed RA-response elements (RARE) located within enhancer regions of RA target genes [140]. In the absence of RA, unliganded RAR-RXR recruits histone deacetylases and nuclear corepressors to inhibit transcriptional activation at the RARE [140,141]. Chondrogenesis requires the absence of RA signalling, in which the repressor function of unliganded RAR-RXR on RAREs dominates [141,142], whilst active RA signalling prevents the chondrogenic differentiation of precursor cells [143]. Crosstalk between the HIF and RAR signalling systems is well documented, but how they co-operate to modulate bone formation is still incompletely understood [144,145,146,147,148]. Due to the pleiotropic function of these pathways, it is anticipated that any therapeutic application to inhibit HO may have off-target effects, as these pathways also dynamically regulate several other critical cellular processes [149].

## 5. Therapeutic Strategies for HO

Treatment strategies for acquired HO to date have included the use of anti-inflammatory agents, bisphosphonates, local radiation therapy, and surgical resection. Systematic reviews have shown that patients treated with either selective or non-selective non-steroidal anti-inflammatory drugs (NSAIDs) showed a significant decrease in post-traumatic HO formation when compared with placebo [150,151,152], but were associated with a higher rate of drug discontinuation due to gastrointestinal side effects. Low-dose local radiation therapy also decreases the incidence of HO after surgery [153,154], but carries the risk of irradiation-induced malignancy [155] and side-effects such as delayed wound-healing, progressive soft-tissue contracture, non-union, and inhibited ingrowth of cementless hip implants [156,157]. The treatment of mature HO after trauma involves surgical resection, although complete excision may not be feasible and recurrence is common [158,159,160]). Simple bisphosphonates, such as etidronate, have also been studied as a prophylactic intervention in HO, as they delay matrix mineralisation. However, bisphosphonates do not inhibit bone matrix synthesis, and mineralisation recommences after drug discontinuation [161,162]. None of these strategies specifically target molecular pathways involved in HO pathogenesis. However, as our understanding of these cells and pathways evolves, molecular mechanism-specific investigative therapeutic approaches are beginning to emerge, as outlined below).

### 5.1. Palovarotene and Other RAR Agonists

The observation that RA signalling suppresses chondrogenesis has stimulated its investigation as a therapeutic target for HO. Synthetic retinoid agonists selective for nuclear RARα or RARγ have been tested in mouse models of injury-induced intramuscular HO, implantation of rhBMP-2 and constitutive activation of mutant *ACVR1* (Q207D) [51,163]. Whilst RA agonists targeting both RARα and RARγ inhibited endochondral HO, those targetting RARγ were most effective as RARγ is more strongly and selectively expressed in chondrogenic cells than other RAR members [164,165]. Chakkalakal and colleagues showed that palovarotene prevented HO, restored long bone growth, and preserved growth plate function in transgenic mice carrying the human *ACVR1* (R206H) mutation for classic FOP [166]. In juvenile FOP mice, palovarotene reduced HO both in vitro and in vivo, but resulted in aggressive synovial joint overgrowth and long bone growth plate ablation [167]. In a rat model of post-traumatic HO (in which rats were subjected to blast overpressure via a shock tube resulting in femur fracture, soft tissue crush injury, and amputation through the zone of injury [168]), Palovarotene treatment suppressed the systemic and local inflammatory response, decreased osteogenic progenitor colonies by 98% in both in vitro and in vivo, and decreased the expression of osteo-and chondrogenic genes, including BMP4 [168]. In another trauma-induced model, rats were subjected to blast-related limb injury, femoral fracture, quadriceps crush injury, amputation and infection with methicillin-resistant Staphylococcus aureus (MRSA) [169]. Palovarotene treatment decreased HO by 50–60%, however 63% of rats treated with palovarotene and inoculated with MRSA experienced delayed healing or dehiscence compared to 25% of MRSA rats in the placebo arm of the study. Palovarotene is currently the subject of several clinical trials of efficacy and safety for the prevention of new HO lesions in both children and adults with FOP (www.clinicaltrials.gov; accessed on 5 May 2022, NCT02190747, NCT03312634, NCT02979769, NCT02521792, NCT05027802). However, whether Palovarotene or other RAR agonists represent a viable approach for treating acquired HO in humans remains unstudied. 

### 5.2. Targeting ACVR1/ALK2 and Other Related Signalling Pathways

Under physiological conditions in normal tissues, the ligand Activin A interacts with ALK2 to mediate SMAD2/3 phosphorylation to regulate cell proliferation, apoptosis, and differentiation (Figure 1) [170,171,172,173,174]. In ALK2R206H+ FOP cells (that carry the common *ACVR1* mutation) ALK2 is activated constitutively in the absence of BMPs, enhancing both canonical and non-canonical BMP signalling pathways [175,176,177,178,179] to augment chondrogenesis [39,179,180,181,182,183]. Although the *ACVR1* mutation is not implicated in other forms of HO, ALK2 signalling has been explored as an investigational target due to its BMP agonism [184]. Table 3 provides a summary of molecular targets and investigational therapeutic strategies explored to date in HO prevention and treatment.

**Table 3:** Summary of investigational therapeutic strategies for the inhibition of heterotopic ossification, based on ALK2 signalling and other pathways. FOP = fibrodysplasia Ossificans Progressiva, tHO = acquired post-traumatic Heterotopic Ossification.

Type of HO Pathways	Type of Molecule	Molecule	Description and Function	Key Papers
	Antibody	REGN2477 (Garetosmab)	Anti-activin-A human monoclonal antibody in phase 2 clinical trial for FOP (LUMINA-1 study, NCT03188666). Blocks signalling of activin A, AB, and AC. Inhibits HO in animal model of FOP.	[179,185,186,187]
FOP	Antibody	Perhexiline maleate (Pex)	Identified in screening of 1040 FDA-approved drugs for suppression of the Id1 promoter activated by mutant ACVR1/ALK2 in mouse C2C12 myoblasts. Pex reduced HO volume in BMP-induced mouse model, but failed to inhibit HO in an open-label clinical trial in FOP.	[188,189]
tHO	Antibody	Metformin	Regulates osteogenic differentiation via AMPK, and RUNX2/CBFA1 in vitro and in vivo. Prevents traumatic HO in mouse by decreasing ALK2 and AMPK regulation of Smad2.	[190,191,192]
FOP	Alpha-2 blocker	Fendiline hydrochloride	Identified in screen of 1040 FDA-approved drugs for suppression of the Id1 promoter activated by mutant ACVR1/ALK2. Mice administered with fendiline showed a slight reduction in HO.	[188]
FOP	Small molecule inhibitor	Dorsomorphin	Identified by chemical library screen for small molecules that dorsalise zebrafish embryos. Selectively inhibited ALK2 to block BMP-mediated SMAD1/5/8 phosphorylation. Preclinical use precluded by the inhibition of other ALKs (ALK3 and ALK6) and other kinases.	[176,193]
FOP, tHO	Small molecule inhibitor	LDN-193189	An optimised version of dorsomorphin with greater potency and selectivity. Inhibits transcriptional activity of ALK2, ALK3, and constitutively active ALK2 mutant proteins.	[124]
FOP, tHO	Small molecule inhibitor	LDN-212854	Derivative of dorsomorphin with increased selectivity for ALK2. LDN-212854 and LDN-193189 reduce osteogenic differentiation of tissue-resident MPCs from injured tissue following burn or tenotomy insult in animal model. In a blast-induced rat tHO model, LDN193189 and LDN212854 effective at limiting tHO.	[194,195]
FOP, tHO	Small molecule inhibitor	Other dorsomorphin derivatives	Currently undergoing investigation, including K02288, DMH-1, ML347, LDN 214117 and VU465350.	[196,197,198]
FOP	Small-molecule inhibitor	Saracatinib (AZD-0530)	Identified by screening compounds in an ALK2-mutated chondrogenic ATDC5 cell line. Inhibited both BMP and TGF-β signalling in vivo. Currently undergoing phase 2 clinical trial for FOP (NCT04307953). Well tolerated and potently inhibits the development of HO in inducible ALKQ207D transgenic and ACVR1R206H knock-in mouse.	[199,200,201,202]
FOP	Small-molecule inhibitor	PD 161570	Identified by screening compounds in an ALK2-mutated chondrogenic ATDC5 cell line. Inhibits both BMP and TGF-β signalling in vivo.	[199]
FOP	Small-molecule inhibitor	TAK 165	Identified by screening compounds in an ALK2-mutated chondrogenic ATDC5 cell line. Indirectly modulates mTOR signalling in vivo.	[199]
FOP	Ligand traps	sActR-IIA-Fc and sActR-IIB-Fc	ACVR1-Fc fusion proteins comprising the extracellular domain of human WT ACVR1 and the Fc portion of human immunoglobulin γ1. Inhibits dysregulated BMP signalling caused by FOP mutant ACVR1 and abrogates chondro-osseous differentiation in vitro.	[203,204,205]
FOP	Platelet inhibitor	Dipyridamole	Identified in screening of 1280 FDA-approved compounds for suppression of ACVR1 gene expression. Showed the highest inhibitory effect on SMAD signalling, chondrogenic and osteogenic differentiation in vitro. Reduced HO in BMP-induced model in mice.	[206,207]
FOP, tHO	Nucleotides	microRNAs	Altered expression of miRNA detected in HO. mir148b and mir365 down-regulate ACVR1/Alk-2 expression, whereas mir26a showed a positive effect on its mRNA. Inhibition of miRNAs, miR-146b-5p and -424 suppresses osteocyte maturation. Manipulating miR-574-3p levels both in vitro and in vivo inhibits chondrogenesis. miR-630 downregulated in early HO and used to distinguish HO from other processes in tHO. miR-17-5p upregulated in ankylosing spondylitis (AS) patients versus non-AS individuals. Knockdown and overexpression of miR-17-5p in fibroblasts derived from AS patients modulates osteogenesis.	[208,209,210,211,212,213,214]
FOP, tHO	Nucleotides	Antisense oligonucleotide (AON)	AON binds to specific exons in the primary mRNA transcript to prevent splicing and enable the skipping of specific exons. AONs designed to knockdown ALK2 expression in mice impair ALK2 signalling in both C2C12 end endothelial cells. However, AON affects both wild-type and mutated allele.	[215,216,217]
FOP, tHO	Nucleotides	RNA interference (RNAi)	Allele-specific siRNA (ASP-RNAi) duplexes tested for specific inhibition of mutant c.617A allele in mesenchymal progenitor cells from FOP patients. ASP-RNAi decreased BMP signalling to control cell levels.	[218,219]
tHO	Nucleotides	LncRNAs	Several lncRNAs regulate bone formation. Downregulation of *MANCR* inhibits osteoinduction in vitro. In a mouse in vivo tHO model, *Brd4*-*Mancr* signalling attenuated HO.	[220,221,222]

## 6. Conclusions

In summary, heterotopic ossification may arise from both rare, heritable and common complex diseases. The downstream molecular pathways that underpin these heterogeneous aetiologies are broadly similar in both patterns of disease, although the diseases differ in extent and severity. Whether genetic or acquired, initiation of a new HO lesion involves tissue injury that results in a signal to initiate endochondral or intra-membranous ossification. The dominant cell types in HO include are FAPs, endothelial cells, hematopoietic cells, tendon and ligament progenitor cells, pericytes and Hoxa11+ mesenchymal stromal cells. The dominant pathways in HO include BMP, mTOR and RAR signalling. Several therapeutic strategies have been developed to target these signalling pathways. RAR agonists have been shown to be effective in preventing HO in pre-clinical models. Although the RAR agonist Palovarotene is undergoing clinical trials for FOP, further pre-clinical animal studies will be required to investigate its efficacy and safety for the post-traumatic HO indication. Several strategies have been developed to target ACVR1/ALK2 with REGN2477, metformin and dorsomorphin derivatives being a few prospects for clinical therapeutic applications. These future studies would benefit from translational experimental approaches that incorporate clinically relevant animal models in parallel with clinical investigations, population epidemiology studies and relevant molecular medicine techniques.

## Data Availability

Not applicable.
